# Life Science’s Average Publishable Unit (APU) Has Increased over the Past Two Decades

**DOI:** 10.1371/journal.pone.0156983

**Published:** 2016-06-16

**Authors:** Radames J. B. Cordero, Carlos M. de León-Rodriguez, John K. Alvarado-Torres, Ana R. Rodriguez, Arturo Casadevall

**Affiliations:** 1 Instituto de Bioquímica Médica (IBqM) Leopoldo de Meis, CCS, Universidade Federal do Rio de Janeiro, Rio de Janeiro, 21941902, Brasil; 2 Department of Microbiology and Immunology, Albert Einstein College of Medicine, Bronx, New York, 10461, United States of America; 3 Department of Neuroscience, Albert Einstein College of Medicine, Bronx, New York, United States of America; 4 Department of Biomedical Engineering, Rutgers University, Piscataway, New Jersey, 08854, United States of America; Universidad de Las Palmas de Gran Canaria, SPAIN

## Abstract

Quantitative analysis of the scientific literature is important for evaluating the evolution and state of science. To study how the density of biological literature has changed over the past two decades we visually inspected 1464 research articles related only to the biological sciences from ten scholarly journals (with average Impact Factors, IF, ranging from 3.8 to 32.1). By scoring the number of data items (tables and figures), density of composite figures (labeled panels per figure or PPF), as well as the number of authors, pages and references per research publication we calculated an Average Publishable Unit or APU for 1993, 2003, and 2013. The data show an overall increase in the average ± SD number of data items from 1993 to 2013 of approximately 7±3 to 14±11 and PPF ratio of 2±1 to 4±2 per article, suggesting that the APU has doubled in size over the past two decades. As expected, the increase in data items per article is mainly in the form of supplemental material, constituting 0 to 80% of the data items per publication in 2013, depending on the journal. The changes in the average number of pages (approx. 8±3 to 10±3), references (approx. 44±18 to 56±24) and authors (approx. 5±3 to 8±9) per article are also presented and discussed. The average number of data items, figure density and authors per publication are correlated with the journal’s average IF. The increasing APU size over time is important when considering the value of research articles for life scientists and publishers, as well as, the implications of these increasing trends in the mechanisms and economics of scientific communication.

## Introduction

The scientific literature is a critical venue for communication of research findings and provides continuity between current and past investigations. In recent years, there have been increasing concerns with the health of the scientific literature [1] including publication biases [2], reproducibility of published work [3,4], increasing number of retractions [5–7], scientific misconduct [8] and mechanisms for correction of erroneous reports [9]. In addition, many of the biological sciences are gripped by an IF mania that often assigns value to scientific work more on the basis of the publication venue than the information content of the study [10]. These problems in conjunction with increasing pressures on science from funding reductions [11] and work force imbalances have led to calls for reforming the scientific enterprise [12–14].

Scientific findings in the biological sciences are usually communicated in the form of written manuscripts (printed and/or electronic) that generally present the new information data in the form of data items (diagrams or figures, tables, and more recently, videos). The typical scientific publication has a formulaic format that combines data items with several written sections, including an introduction, followed by methods, results, and discussion.

The size and type of scientific articles can differ significantly from short notes to long papers depending on the area of study and the restrictions of the publication venue. Prior to publication, the information content published by scholarly journals normally passes through peer-review by experts within the scientific community. Yet, this process is associated with hidden costs estimated in the billions pounds per year [15]. The peer-review process represents a rate-limiting step in scientific communication, providing a valuable checkpoint for the logic, validity, reliability and novelty of scientific claims [16].

As the size of the published literature continues to increase [17–22], the ability to review, manage, disseminate and integrate scientific information appropriately remains an important concern. The paper inflation phenomena that began between 1960s and 1980s was attributed to the increasing number of authors and the publication of shorter articles [23]. This practice gave rise to the Least Publishable Unit or LPU, a concept that acquired a negative connotation since it is often seen as the result of packaging scientific findings into smaller articles units (‘salami slicing’) with the goal of achieving more publications from a research project [23]. The pros and cons of such publication practices remain a controversial topic, and since the inclusion of supplemental sections in the early 2000s, the size of publications in some journals has increased [24,25]. However, evidence for or against a general trend towards increasing information content per publication in the biological literature is still lacking. Considering the importance of research articles for scientists and publishers, estimating the amount of information that equates to a publication and how it changes over time is important in the study of the mechanisms and economics of science.

The amount of information contained in a research article has been previously measured by estimating the publication’s size, in terms of pages, bytes and/or number of figures and tables [23,25–27]. Estimations based on number of pages and/or bytes are subjected to possible changes in journal manuscript formats (eg., font size, margins, page limitations, figure size) and are limited by the fact that textual descriptions, data tables, and image resolutions can vary substantially in length and/or file size without a change in information content. We propose that the number of data items and density of composite figures are good parameters for determining the change in information content per research article over time. Our reasoning considers that (1) figures and tables are central to research articles in disciplines related to the biological sciences, containing the bulk of the new information, (2) each figure or table almost always equates to at least one new claim, and (3) multipanel or composite figures are often used to group multiple pieces of evidence in support of at least one new claim.

In this study, we measured the APU (that can be defined as the average amount of scientific content that equates to a scholarly publication) in 1993, 2003, and 2013 from 10 leading journals in the biological sciences. Our findings suggest that the average number of data items and panels per figure (PPF) ratio per article have approximately doubled over the past 20 years of life science publishing. As expected, the increase in data items per publication was mainly in the form of supplemental material (SM). The average number of pages, references and authors per article also increased significantly in the past two decades. The average number of total data items, PPF ratio and authors per publication correlated with journal’s average IF. The data presented here may facilitate others’ investigations of the economics and effectiveness of scientific publishing and communication. We discuss possible implications of these trends in the process of science, the scientific literature and the peer-review process.

## Materials and Methods

A total of 10 indexed scientific journals publishing Research Articles in the life-sciences were examined; having different IF for the three years studied [1993, 2003, 2013, respectively]: (*Biochemistry* [5.11, 3.92, 3.37], *Journal of Bacteriology* [3.97, 4.18, 3.18], *Journal of Biological Chemistry* [3.79, 6.48, 4.77], *Journal of Immunology* [7.07, 6.70, 5.36], *Journal of Neuroscience* [8.04, 8.31, 7.12], *Journal of Virology* [5.65, 5.23, 5.08]), *Cell* [37.19, 26.63, 32.40], *Nature* [22.32, 30.98, 38.60], *Proceedings of the National Academy of Sciences (PNAS)* [10.33, 10.27, 9.74], and *Science* [21.70, 29.16, 31.20]). The IF is a numerical measure for ranking journals indexed by Thomson Reuters and is based on the ratio of total number of citations in the current year of any items published in the previous two years to the total number of certain publications (i.e., research articles, reviews, notes) during the same period [28].

A sample size, *n*, of 50 articles per year (1993, 2003, and 2013) for each journal was considered because it is a practical number to be analyzed manually across journals and based on the assumptions that the number of data items per publication of an unknown population size exhibited a normal distribution and a narrow standard deviation, s = 1, from the mean, regardless of the year. Based on the equation: *n* = (*st*/*ME*)^2, these assumptions would yield means approximations with a 95% (*t*-score = 1.96) confidence interval (or margin of error, *ME*) of 0.28 (since we assumed such a narrow *s*). However, the observed 95% *MEs* of sample means±sd among journals obtained from our analysis ranged from approximately 0.13–1.2 in 1993, 0.3–1.9 in 2003, and 0.8–6.0 in 2013.

Papers relevant to the biological sciences were selected by personal assessment of article’s title, abstract, keywords and data items. For consistency across journals, this study only considered publications classified by each journal as Research Articles and not other publication types such as letters, reports, reviews, etc. Research Article size restrictions (S1 Table) show little consistency among journals, varying in terms of number of words, characters, pages, data items and/references. Articles were selected chronologically starting from October issues, a month chosen simply because this study began in October 2013. For journals that publish more than 50 Research Article manuscripts types per month, the most recently entered citations retrieved from PubMed (MEDLINE database) were selected. For journals that publish less than 50 articles per month, additional articles published chronologically in following months from the same year were included to meet our *n*. Given that some journals such as *Nature* and *Science* only publish a few Research Articles per issue related to the biological sciences (most publications are classified as Letters or Reports, respectively) and did not reach 50 articles per year, the total number of articles examined was 1464 instead of the expected 1500. Although we do not expect that publication content was somehow influenced by the months of the year, the arithmetic means of 50 research articles published in October and May of 2013 for the same journal (PNAS) were compared to check how the number of data items and pages per article varied with the month of publication. As expected, no statistical difference (p = 0.3 and p = 0.9, respectively) when the two months were compared by *t*-test and Kruskal-Wallis test.

The discrete variables determined for each research article and corresponding online SM (when present) included the total number of: (*i*) data items (figures, tables); (*ii*) labeled PPF; (*iii*) pages; *(iv)* cited references; and (*v*) authors. Quantification of total data items, PPF ratio and references was done by manual inspection. The criteria for counting the number of panels inside figures was based on the letter/number code or labeling style in order to maintain consistency and unbiased evaluation among inspectors. The clustering and/or labeling of panels vary between data types and authors in two styles: *i*. when two panels are labeled as one (e.g. control + treatment = [panel A]), versus *ii*. labeling each panel (e.g. Control [panel A], treatment [panel B]); the prior being the predominant labeling style in our sample. In these two examples the ratio would be counted as 1 and 2, respectively.

The number of authors and pages per article was determined from the abbreviated descriptions of Research Article lists retrieved from PubMed as a CSV (comma-separated values) file. Author number was determined using the SPLIT and COUNTBLANK spreadsheet functions. The articles that listed the *et al*. (abbreviation for “and other” authors) were inspected manually to obtain the total number of authors. The page number of each article was determined from the page range abbreviation. The page range abbreviation omits the common leading digits of the end-page. To generate the full digit end-page value, the IF, LEN, and REPLACE functions were used. The total number of pages per article was calculated by subtracting the value of the start page from the end page and adding 1. Data collection, functions, and arithmetic operations were done using the Spreadsheet Google Drive software. Statistical analyses were performed using Prism version 5.0b (GraphPad Software, San Diego, CA).

## Results

**Fig 1:**
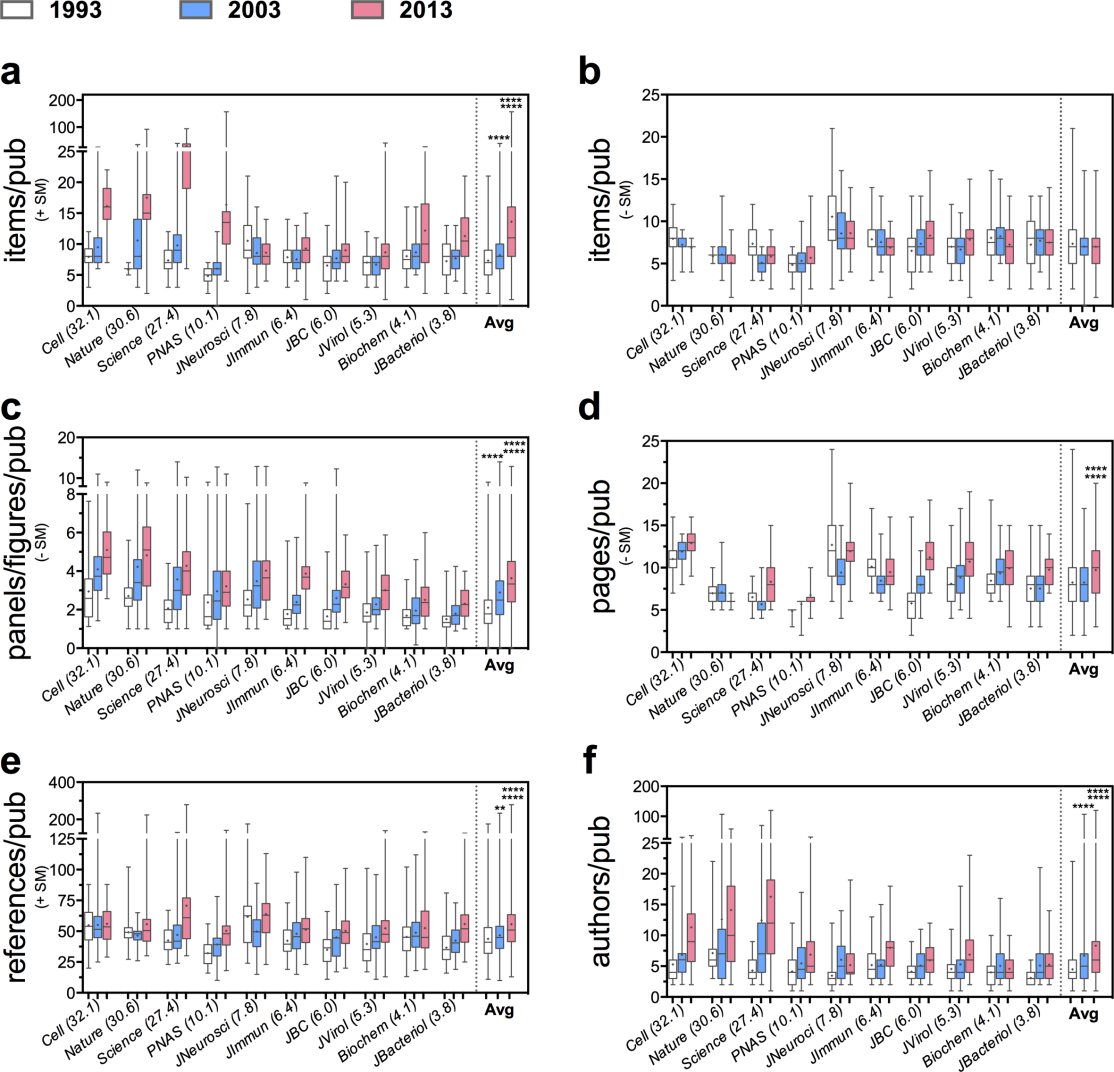
Increase in the Average Publishable Unit (APU) over the past two decades of scientific publication. A total of 1464 research articles were visually-inspected to quantify: the average number of (**a**) total data items (including supplemental material, +SM); (**b**) data items in main article alone (-SM); (**c**) PPF ratio or panels inside composite figures (-SM); (**d)** pages (-SM), (**e**) references (+SM), and (**f**) authors per publications in 1993 (white), 2003 (blue), and 2013 (red) by 10 scholarly journals with diverse average IF (in parenthesis). Box plots display the average median (horizontal line in the box interior), interquartile range or distance between the 25^th^ and 75^th^ percentiles (box limits), and the minimum and maximum values (vertical lines issuing from the box) for each variable measured. The mean is indicated by the “+” symbol inside the box interior. Averages considering all articles (487 articles for 1993, 484 for 2003, and 493 for 2013) from all journals are shown on the right-end (note divisor line). Kruskal-Wallis non-parametric statistical test: *P≤0.05; **P≤0.01; ***P≤0.001; ****P≤0.0001. For the year 2013, top and bottom asterisks correspond to statistical analysis between 2013–1993 and 2013–2003, respectively.

**Fig 2:**
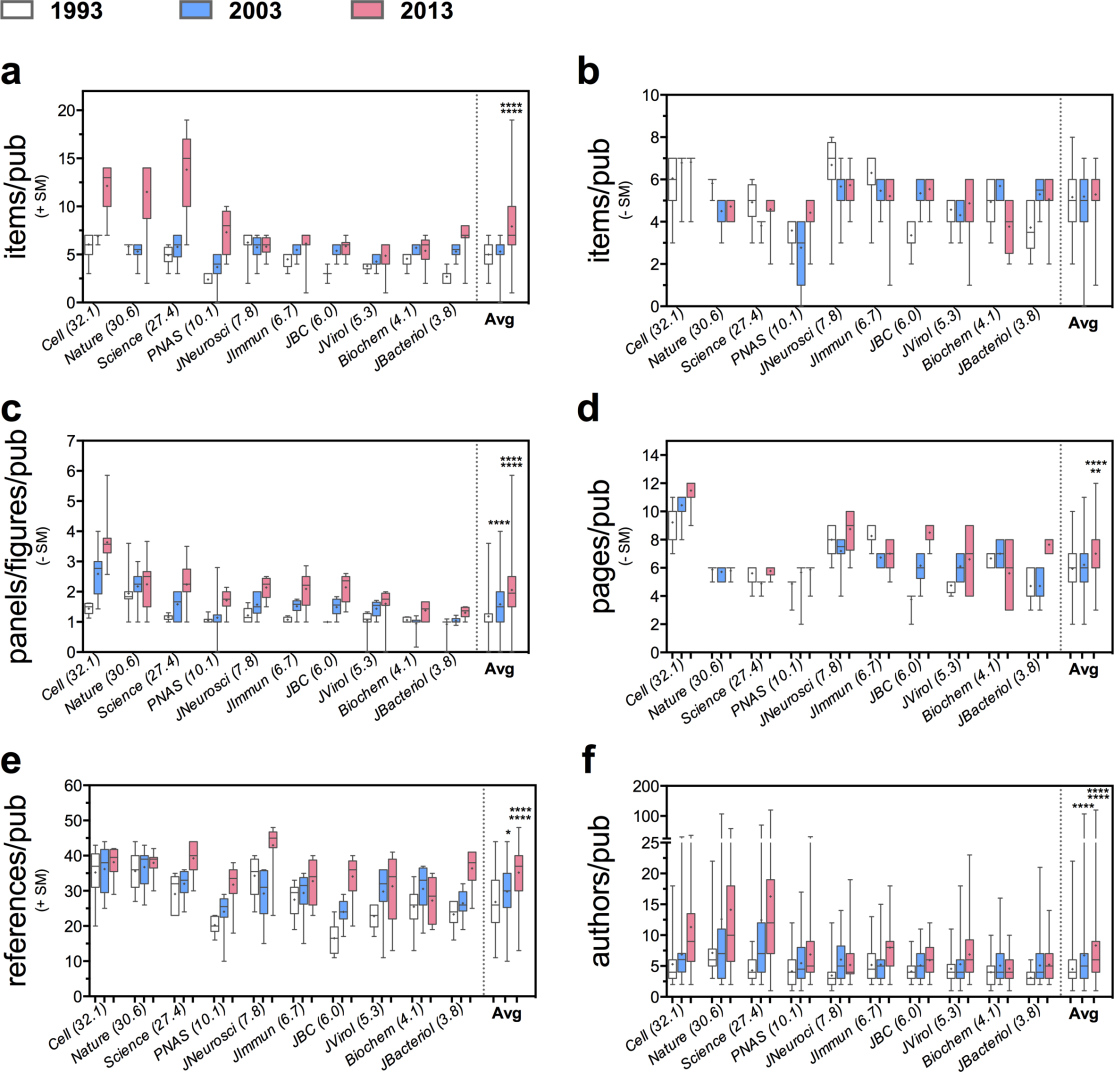
Change in the average first-quartile publishable unit (AQ1PU) over the past two decades. Box plots showing the change in the lower quartile group (25^th^ percentile and less) of the APU as shown in Fig 1.

To investigate how the biological literature has changed during the past twenty years of scientific publishing, we monitored a series of discrete continuous variables (data items, the PPF ratio, pages, references and authors) for 1464 research articles and SM published in 1993, 2003, and 2013 by 10 leading scientific journals with different IFs. The data for all variables exhibited non-normal distributions. With the exception of data items in main article (not including SM), all variables showed a general increase in the average, variance, and first quartile (Q_1_) over time (Figs 1 and 2).

Based on the sample, the average amount of data items per research article, including SM, approximately doubled, from 7±3 to14±11, over the past two decades (Fig 1A). From highest to lowest, the percentage mean difference from 1993 to 2013 for each journal was: 298% for *Science*; 238% for *PNAS*; 200% for *Nature*; 103% for Cell; 56% for *Journal of Bacteriology*; 52% for *Biochemistry*; 38% for *Journal Biological Chemistry*; 24% for *Journal of Virology*; 18% for *Journal of Immunology*; and -18% for *Journal of Neuroscience*. *Cell* and *Nature* showed a significant increase in data content (20 and 81%, respectively) already in the period of 1993 to 2003. *Science* and *PNAS* showed large increments of at least twice the average amount of data items per publication from 2003 to 2013.

The overall increment of data items per research article is in the form of SM, since the average number of data items contained in the main article (not including SM) did not vary significantly (Fig 1B) from 1993 to 2013: 7±3 to 7±2. Six out of 10 journals showed an average increase (4 to 27%) while four showed a decrease (-10 to -21%); averaging an overall decrease of ~5% data items per article over the past two decades. On average, the amount of data items in the SM represented 58–80% of the total for journals with average IF>15 and 0–40% of the total for journals with average IF<15. Among the journals analyzed, the *Journal of Neuroscience* was the exception given that it first included supplemental section in 2003 and discontinued it in 2010 [25]; thus, it showed a -18% relative change in the average number of data items per article, which remained relatively constant since 2003.

A second measurement for information density was derived from the number of figure panels divided by the number of figures in the main article (Fig 1C). Overall, the PPF ratio also approximately doubled from 2±1 to 4±2 between 1993 and 2013, and visually resembled a Poisson distribution model. The increase in variation of the PPF ratio was less pronounced (two-fold standard deviation increase) than that of the average number of data items per article, which increased more than three-fold in the past two decades (Fig 1A). From highest to lowest, the average percentage increase from 1993 to 2013 for each journal was: 118% for *Journal of Immunology*; 106% for *Science*; 101% for *Journal of Biological Chemistry*; 78% for *Nature*; 73% for *Cell*; 62% for *Journal of Virology*; 59% for *Journal of Neuroscience*; 53% for *Journal of Bacteriology*; and 48% for *Biochemistry*; and 35% for *PNAS*.

The overall average main article page length also increased significantly (approximately 18%) from 8±3 to10±3 between 1993 and 2013 (Fig 1D). Seven journals showed a significant increase in average page number (94% for *Journal of Biological Chemistry;* 32% for *Journal of Virology*; 39% for *Proceedings of the National Academy of Sciences*; 29% for *Journal of Bacteriology;* 28% for *Science*; 17% for *Biochemistry;* 16% for *Cell*) while three journals showed a decrease (-6% for *Journal of Immunology* and *Journal of Neuroscience;* -14% for *Nature*).

The overall average number of references, (including SM), showed a significant increase (~27%) from 44±18 to 56±24 per unit publication from 1993 to 2013 (Fig 1E). From highest to lowest, the percentage increase of the mean from 1993 to 2013 was: 67% for *Science*; 56% for *PNAS*; 52% for *Journal of Bacteriology*; 44% for *Journal of Biological Chemistry*; 32% for *Journal of Virology*; 21% for *Journal of Immunology*. The changes in mean number of references for the journals *Biochemistry*, *Nature*, *Journal of Neuroscience*, and *Cell* were not significant; with 16, 14, 4, and 2%, respectively.

The overall average number of authors per article also increased significantly from 5±3 to 8±9 from 1993 to 2013 (Fig 1F). From highest to lowest, the percentage increase of the mean from 1993 to 2013 was 280% for *Science*; 114% for *Cell*; 98% for *Nature*; 70% % for *Journal of Bacteriology*; 66% for *PNAS*; 54% for *Journal of Immunology*; 50% for *Journal of Neuroscience*; 52% for *Journal of Virology*; 44% for *Journal of Biological Chemistry*; and 14% for *Biochemistry*.

We also examined the changes in the left tail of the distribution for all article characteristics by comparing the first quartile (Q_1_) group of values from each journal and year (Fig 2). That is, we compared values corresponding to the 25^th^ percentile or bottom 25% from each journal for each of the variables measured. Including all journals from 1993 to 2013, the average 25^th^ percentile number of data items per article increased 59% (including SM) and 2% (not including SM) (Fig 2, panels a and b); PPF ratio increased 71% (Fig 2C); pages increased 18% (Fig 2D); references increased 31% (Fig 2E); and authors increased 30% (Fig 2F). These results suggest that the frequency of smaller size publications is gradually shifting towards larger ones.

**Fig 3:**
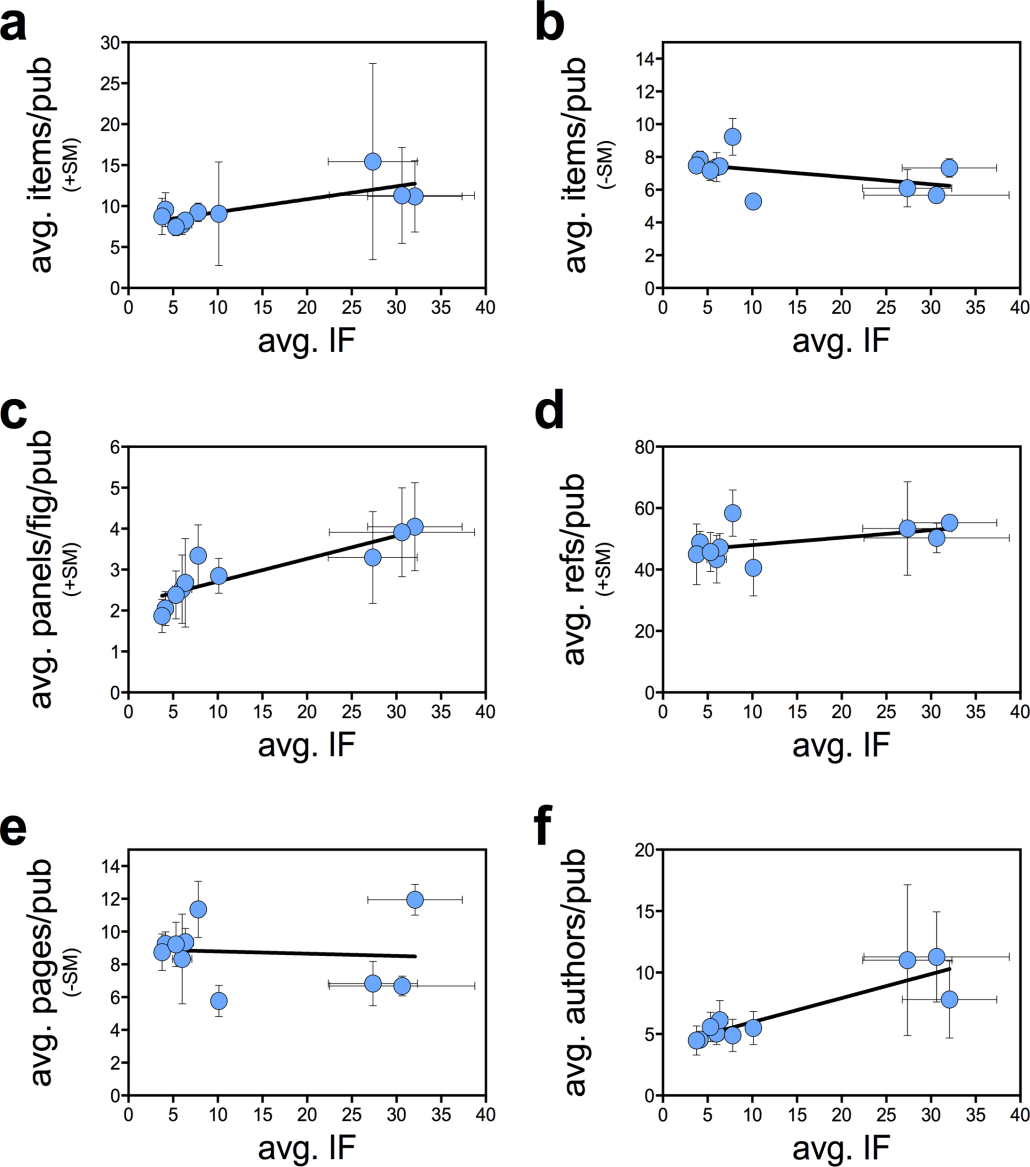
Correlation between the APU variables and journal’s average IF. Linear regression and Spearman’s rank-order correlation (Spearman’s critical rho (N = 10, alpha = 0.05) = 0.648) analysis of average IF and average number of (**a**) total data items (including supplemental material, +SM) (R^2^ = 0.14, p = 0.04; Spearman’s rho = 0.65, p = 0.05): (**b**) data items in main article alone (-SM) (R^2^ = 0.17, p = 0.03; rho = -0.56, p = 0.10); (**c**) PPF (R^2^ = 0.40, p = 0.0002; rho = 0.96, p<0.0001); (**d**) pages (R^2^ = 0.01, p = 0.71; rho = -0.08, p = 0.84); (**e**) references (R^2^ = 0.10, p = 0.08; rho = 0.50, p = 0.14); and (**f**) authors (R^2^ = 0.44, p<0.0001; rho = 0.81, p = 0.007) per publication. Error bars represent standard deviations.

Both linear regression and non-parametric Spearman’s rank-order correlation analyses yielded significant (Spearman’s critical rho (N = 10, alpha = 0.05) = 0.648) correlations between the journal’s average IF and the average number of data items (including the SM) (R^2^ = 0.14, p = 0.04; Spearman’s rho = 0.65, p = 0.05) (Fig 3A), the average PPF ratio (R^2^ = 0.40, p = 0.0002; rho = 0.96, p<0.0001) (Fig 3C) and the average number of authors (R^2^ = 0.44, p<0.0001; rho = 0.81, p = 0.007) (Fig 3F) per publication. Analysis between the journal’s average IF and the average number of data items (not including the SM) (R^2^ = 0.17, p = 0.03; rho = -0.56, p = 0.10) (Fig 3B), the average number of references (R^2^ = 0.10, p = 0.08; rho = 0.50, p = 0.14) (Fig 3D) and the average number of pages (R^2^ = 0.01, p = 0.71; rho = -0.08, p = 0.84) (Fig 3E) per publication showed no correlation by one or both tests. When combining all three publication years, a comparison of high-IF (>15) versus low-IF (<15) journals showed clear distribution differences for all variables: data items ±SM (Mann-Whitney U test, p<0.0001), PPF ratio (p<0.0001), references (p<0.0001) and authors (p<0.0001), with the exception of pages per publication (p = 0.1791). Notably, in 1993, when the SM section was unconventional, the average number of data items per publication between high-IF (>15) and low-IF (<15) journals showed no statistical difference (p = 0.648).

The number of total data items (including SM) correlated with the number of authors in a publication (Spearman’s rank correlation: rho = 0.1, p = <0.0005), particularly for high-IF (>15) journals (rho = 0.4, p = <0.0001). Overall, the average proportion of data items per authors per publication was approximately 2.2±1.2, 1.9±1.3, 2.3±1.9 for 1993, 2003, and 2013, respectively.

In summary, our data shows that the APU has significantly changed during the past two decades and that in 2013, the APU for the sampled population consisted of approximately 14 data items (+SM), 4 panels per figure ratio (-SM), 10 pages of length (-SM), 56 references (+SM) and 8 authors.

## Discussion

In this study, we document an increase in the information density for published papers in the biological sciences over the past two decades by calculating the number of data items and PPF ratio for 1464 research articles. These measurements are part of what we define as the average publishable unit, or APU, which represents the typical information content of a published research article. The average number of data items (including SM) and average number of PPF ratio (in the main article alone) per publication doubled in the past two decades. Concomitantly, we measured an increase in the average number of pages, authors, and a modest change in reference citations per article over time. The strong monotonic correlations observed for our sample population suggests that journals with higher IF tend to publish research articles with higher number of data items, figure density and authors relative to the lower IF journals. However, we cautious that this is preliminary finding and we require addition analysis before firm conclusions could be made. Although these measurements correspond to a restricted sample population and should not be generalized to all manuscript types, scientific disciplines and/or journals, these trends in the scientific literature are important because they could potentially reflect major changes in the practice and culture of science as a whole.

The publication of scientific findings is generally considered an essential step in the completion of a research project. The costs associated with research production and communication is substantial, involving apparent cash costs in the form of salaries, supplies, and cutting-edge technologies, as well as other indirect and hidden costs (e.g. facilities, administrative and peer-revision of articles by experts) [15,29–31]. Although voluntary, the hidden costs associated with peer-review were recently estimated in the billions pounds per year; representing 29% of global costs related to publishing and distribution of journal articles [15]. An increase in information content per publication could mean increasing work for reviewers who, in addition to coping with increasing numbers of manuscripts, must also meticulously inspect the material to insure the integrity of the publication process. Increasing reviewer workload could translate into information overload for reviewers and a reduction in the quality of the peer-review process. Even though reviewers are not remunerated for this work, the opportunity costs associated with the time spent on proper [16,32,33] peer-review should increase with greater data density per publication. Since publication in peer-reviewed scholarly journals is the primary form of disseminating scientific information and peer-review process is a key rate-limiting step in this process, a rising information density per publication could suggests that completion of scientific work is significantly more costly now than two decades ago.

The increase in information density per article is likely to have multiple causes. First, the proliferation of technologies in the past two decades have allowed investigators to probe scientific questions more deeply, introducing new forms of data. For instance, biological techniques such as genomics, proteomics, glycomics, etc., commonly referred to as the “omics”, have become accessible to more laboratories in recent years as fee-for-service basis. Along with informatics, these technologies usually generate large volumes of data that require additional figures and inclusion in supplementary material sections. Second, many scientific studies are now multidisciplinary [34]. In the past scientific papers dealt with one facet of a problem (e.g. biochemistry, cell biology, molecular biology, etc.) while today many publications include multiple pieces of data that were formerly in the domain of each of these disciplines. Adding to this trend is the emphasis on translational science, which often requires the addition of clinical material to basic scientific studies to achieve the goal of “translation”. Third, as some investigators adopt these techniques they change the standards in the field for what is needed for publication, similar to a herd behavior [35,36], and thus, raise the bar for other investigators aiming to publish in the same types of journals. This in turn drives the bar higher for subsequent work. Fourth, the proliferation of personal computers and the ease with which new figures can be generated, together with a new plethora of schemes by which data can be presented (e.g. scatter plots) could be encouraging authors to include more data in new formats that were not easily available in the past. Lastly, reviewers may be requiring more data to recommend acceptance of manuscripts. In this context, Ploegh has argued that reviewers increasingly demand additional experimental work during the peer-review process in a practice that he called a “wasteful tyranny” [37]. These forces are not probably independent but together could synergistically stimulate the growth of information density per article. We also considered the possibility that journal guidelines and/or possible changes in article format were contributing to the increase in information density but a review of author instructions revealed no evidence for changes that could explain the observed trends. In fact, the general format of the papers published by these journals remained remarkably consistent over the two decades that spanned the time of this study.

The association between information density, both in terms of number of data items and PPF ratio, and the IF of the journal where the work was published raises the possibility that current IF mania gripping the biological sciences [10] is contributing to this phenomenon. Although correlation is not causation we can imagine how current pressure to publish in high impact journals could be contributing to this effect: (1) authors may be packing more information into their studies with the hope that higher information density translates into a higher likelihood for acceptance into the most desirable journals, (2) the most exclusive journals may be demanding more information for publication in an effort to achieve a more comprehensive study that is more likely to be subsequently cited and thus maintain their high IFs. Irrespective of whether such trends are good or bad for science the correlation between information density per paper with journal IF suggests that increasing paper size may be another mechanism by which the IF mania could be distorting the process of science.

Concomitantly, with the increase in information density we note a significant increase in the number of authors per article that also correlated with the average IF of the journal. Since the famous de Solla Price predictions [38], trends toward an increasing number of authors per publication have been widely documented [23,39–44]. Such a trend of increasing collaboration could be explained by the causes suggested above for the growth of information density. The costs associated with the generation of cutting-edge scientific information, the funding restrictions, and the associated risks in scientific publishing in a “winner-take-all” reward system [45] may motivate scientists to team-up, pool resources and fractionate the risks through co-authoring. Also, the increasing complexity of scientific research has resulted in greater specialization of scientists [46], which in turn suggests that the inclusion of additional techniques requires the recruitment of additional investigators to provide that data and thus serve as co-authors. This trend could have both positive and negative consequences. Increased interaction between scientists in diverse fields could translate into greater communication and the possibility for advances at the interfaces of different disciplines. On the other hand, an increase in the number of authors, some of whom bring highly specialized knowledge, could result in reduced supervision of larger groups, and less responsibility per author for the final product and reduced integration of data. The growth in authors brings with it the concerns about the possibility that as more authors are added, there is an increased likelihood of some individuals with reduced integrity and capable of misconduct joining the group. In this regard, we note that the inclusion of one individual who has been accused of misconduct in numerous studies has led to dozens of retractions of scientific publications [47].

One variable in which we observed relatively little growth was the number of literature citations per article. Our results are consistent with previous studies also showing increasing trends over time in the number of references per publication across different fields [18,26,27,48–51], although decreasing trends have also been documented [49]. However, the small increase in this parameter is paradoxical given the increasing size of the scientific literature [18,21,22] and the need for referencing the increasing number of techniques used in each article. The small growth in references per publication agrees with the fact that journals can restrict the number of references by limiting the amount of characters, words and/or pages of articles. Given the other trends that should have pressured an increase in citation number [52] and thereby positively impacting journal IFs, the small increase of this parameter is not understood and requires further analysis. Other possible contributing factors here may include a preference for citing reviews over primary sources and the possibility that under-citation of relevant literature is purposefully used in a conscious or unconscious strategy to enhance the novelty of the reported findings. Whatever the mechanism(s) responsible for this constancy discordance between the number of article references and the size of the literature it could have the detrimental effect of reducing connectivity between what is known and what is being reported.

Given the critical role of the published literature in the maintenance and advance of the scientific enterprise, changes in the literature’s informational content, as well as the causes of such changes, are relevant to the scientific community. In recent years, the quality of the scientific literature has been questioned by an increasing number of retractions [6,7], the finding that most retractions are due to misconduct [7,53] and the realization that many published studies are not reproducible [3,4]. Although these problems are not immediately linked to increasing publication size we can see some connections that suggest avenues for future study. The growth of information density suggests that there is more experimental work per paper and given that any experiment carries a potential for error, increasing the amount of data should increase the potential for error or information noise to be revised during the peer-review process. Errors in one component of a publication could undermine the value of a study and lead to a retraction or subsequent lack of reproducibility if that component is important to the study’s conclusions.

The increasing size of the SM for scientific papers also has important implications for journals and publishers. Since the early 2000’s, the inclusion of SM sections allowed electronic access and relatively low cost storage of more information. However, the abundance of SM has urged some journals to modify their policies [24,25]. For example, after agreeing to publish supplementary data in 2003 *The Journal of Neuroscience* discontinued this practice in 2010 [25], which explains its constant average information content per publication between 2003 and 2013. This decision was motivated by an alarming increase in supplemental file size (bytes) over a seven-year period [25]. The journal recognized that such an increase could negatively affect the peer-review process by encouraging the inclusion of more data requested by reviewers, creating an arms-race between reviewers and authors, and by the segregating information that could affect the communication of important findings [25].

In summary, our study documents a change in the literature of the biological sciences toward publications with more data over time. The causes for these trends are complex and probably include increasing experimental options and changes to the culture of science. At first glance, this data could be interpreted as a cultural change opposite to data fragmentation practices. However, it is also possible that an increase in publication density can still occur over a ‘salami slicing’ culture if the publication unit to be segregated is larger to begin with, as the result of technological improvements and increasing numbers of scientific authors. The benefits and debits of this trend for the scientific process are uncertain at this time but it is clear that there have been major changes to the nature of scientific publications in the past two decades that are likely to have major repercussions in all aspects of the scientific enterprise. High-throughput quantification of the APU using algorithm-based methods are necessary to evaluate a larger number of journals, to better evaluate the temporal behavior of publication density over time and to perform robust regressions and prediction of trends. The patterns of the APU among different subfields in the biological sciences, as well as the relationship between the publication density and citation index are also interesting subjects for future studies. The estimations of information content per publication presented here provide additional parameters to study the evolution, mechanism and economics of scientific communication in the life sciences.

## Supporting Information

S1 Table Research article size restrictions. (DOC)
