# Supplementary material for: Life Science’s Average Publishable Unit (APU) Has Increased over the Past Two Decades
Source: PLoS One. 2016 Jun 16;11(6):e0156983. doi: 10.1371/journal.pone.0156983 (PMC4911092; doi:10.1371/journal.pone.0156983)
Supplement: S1 Table — (DOC) [file pone.0156983.s001.doc]

**S1 Table.** Research Article size restrictions in terms of number of words, characters, pages, data items, and/references (refs) for the journals and publication years included in this study.

| **Journal** | **Size Restrictions of main article** | | |
| --- | --- | --- | --- |
|  | **1993** | **2003** | **2013** |
| *Cell* | 8 data items | NA* | 55,000  12 pages double space, 7 data items |
| *Nature* | 5 pages, 3000 words (not including figure legends), 6 data items, 50 refs | 5 pages, 50 refs | 5 pages (3000 words), 5-6 data items, 50 refs |
| *Science* | 4000 words or 4 printed pages | ~5 pages, 6 data items, 40 refs | ~4500 words including refs, or ~5 pages, 40 refs |
| *PNAS* | 5 pages or 6000 words | 6 pages, up to 49,000 characters | 6 pages, up to 49,000 characters |
| *J Neurosci* | NS | NA | 2150 words including citations |
| *J Immun* | NS | 8 printed pages, 64,000 characters | 64,000 characters |
| *JBC* | < 6 pages (24 double spaced pages including figures & tables | NA | 10 Mega bytes as *PDF* format |
| *J Virol* | not specified | NA | NS |
| *Biochem* | ~6 printed pages including refs | no limits | NS |
| *J Bacteriol* | NS* | NS | NS |

*NA, not available.

*NS, not specified. Instructions for authors did not specified size limitations.

The information was taken directly from printed issues, journal’s website and/or direct email communication with editorial team.
